# Development and validation of a machine learning model to predict prognostic outcomes in infantile epileptic spasms syndrome

**DOI:** 10.3389/fped.2026.1777561

**Published:** 2026-04-07

**Authors:** Caoxue Zuo, Boen Xue, Xiaofeng Mu, Zhongqiang Cao, Huamin Zhou, Zhisheng Liu, Dan Sun

**Affiliations:** 1 Department of Neurology, Wuhan Children’s Hospital, Tongji Medical College, Huazhong University of Science and Technology, Wuhan, China; 2 State Key Laboratory of Material Processing and Die & Mould Technology, School of Materials Science and Engineering, Huazhong University of Science and Technology, Wuhan, China; 3 Wuhan Children’s Hospital, Tongji Medical College, Huazhong University of Science and Technology, Wuhan, China; 4 Institute of Maternal and Child Health, Wuhan Children’s Hospital (Wuhan Maternal and Child Healthcare Hospital), Tongji Medical College, Huazhong University of Science and Technology, Wuhan, China

**Keywords:** infantile epileptic spasms syndrome (IESS), machine learning, model interpretability, predictive model, prognosis

## Abstract

**Objective:**

To develop and validate a machine learning (ML) model for predicting seizure outcomes in infants with infantile epileptic spasms syndrome (IESS).

**Methods:**

This retrospective study enrolled pediatric patients diagnosed with infantile epileptic spasms syndrome (IESS) from Wuhan Children's Hospital. The cohort was randomly split into training and validation sets at a 7:3 ratio. Independent prognostic factors were identified using Cox regression analysis. Six machine learning algorithms were then applied to develop predictive models. Model performance was evaluated in terms of discrimination (e.g., AUROC), calibration (calibration curves), and clinical utility (decision curve analysis, DCA). The optimal model (XGBoost) was interpreted via decision tree visualization and SHAP analysis.

**Results:**

Poor seizure outcome was observed in 56% of the cohort. MRI findings of tuberous sclerosis complex or malformations of cortical development were independent risk factors. Among the models, XGBoost demonstrated the best overall performance, achieving an AUROC of 0.921 in the validation set, along with robust calibration and clinical utility.

**Conclusion:**

The developed ML model reliably and interpretably predicts poor seizure outcomes in IESS patients using routine clinical data, potentially aiding in clinical decision-making and follow-up planning.

## Highlights

- Presents an interpretable machine learning model to predict seizure outcomes in Infantile Epileptic Spasms Syndrome.
- The XGBoost model demonstrated high predictive accuracy (AUC: 0.812) upon validation.
- Identifies key prognostic factors, including medication load and specific MRI abnormalities.
- Offers a practical tool for early risk stratification to guide clinical decision-making.

## 1 Introduction

Infantile Epileptic Spasms Syndrome (IESS) is a severe developmental and epileptic encephalopathy of infancy, often categorized as refractory epilepsy. With an incidence of approximately 30 per 100,000 live births, it is characterized by early onset, substantial neurodevelopmental impairment, and typically poor prognosis. Prognosis in IESS involves several factors, with the most critical being the complete cessation of spasms and subsequent improvements in development and cognition (1). A systematic review by Elysa Widjaja et al. highlighted that neurodevelopmental outcomes in children with IESS are frequently suboptimal (2, 3). Nevertheless, a significant proportion of IESS patients can achieve spasm freedom with effective anti-seizure medications (ASMs). The cornerstone of treatment for IESS is the early and rapid control of epileptic spasms. Prompt cessation of spasms and alleviation of hypsarrhythmia are vital, as these are expected to improve cognitive outcomes (4), thereby improving long-term prognosis. Consequently, seizure outcomes in children with IESS are pivotal in understanding their long-term prognosis.

Numerous risk factors have been identified as significantly associated with IESS outcomes, including etiology (5), treatment timing (6), pre-onset developmental delay (7), abnormal EEG background and features (8), neuroimaging abnormalities (9), and a favorable response to adrenocorticotropic hormone (ACTH) therapy (10). However, most existing studies primarily focus on correlations between clinical variables and neurodevelopmental outcomes. Research integrating clinical factors specifically related to seizure outcomes in IESS remains limited. A comprehensive understanding of these factors would provide a foundation for exploring spasm prognosis and aid in the development of predictive models for outcomes in IESS patients. For example, Yuto Arai et al. (6) identified key predictors for both seizure and developmental outcomes in IESS and developed prognostic models using variable selection and linear/logistic regression. Thus, a thorough investigation of risk factors influencing seizure prognosis in IESS is essential to identify high-risk individuals for poor outcomes and facilitate earlier intervention where possible.

Machine learning (ML) technology utilizes algorithms derived from large datasets to make inferences about future data, enabling the identification of individuals at high risk for specific diseases. With the rapid advancement of artificial intelligence (AI), ML has become increasingly applied in the field of epilepsy (11), particularly in the diagnosis and prediction of seizures (12, 13). In a pioneering study, An et al. (14) first used the Random Forest algorithm to predict the likelihood of drug-resistant epilepsy at the time of ASM prescription. Another study combined six common ML algorithms with multimodal clinical data to develop and validate an effective predictive model for medication outcomes in tuberous sclerosis complex (TSC)-associated epilepsy (15). Despite these advances, few domestic studies have applied ML algorithms to develop predictive models for seizure outcomes in IESS. Internationally, Lucasius et al. (16) utilized a biomimetic deep learning network algorithm with electroencephalography (EEG) data to predict the onset of epileptic spasms in IESS patients. Similarly, Ge (17) employed multiple ML models to process multimodal clinical data from IESS patients and develop a predictive tool for their outcomes. This study aims not only to explore independent risk factors for seizure prognosis in IESS but also to integrate ML algorithms with multimodal clinical data to create a high-accuracy predictive model for IESS outcomes.

This study applied ML algorithms, based on a stable existing clinical database, to develop and validate a predictive model for children with IESS. This model aims to predict the risk of recurrent epileptic spasms in IESS patients at an early stage, alerting pediatric neurologists to identify high-risk children. The ultimate goal is to enable more informed treatment strategies, focusing on early control and cessation of spasms, thereby improving the quality of life for these children. Furthermore, such a tool could optimize healthcare resource allocation and reduce the societal burden associated with this specific patient population.

## 2 Materials and methods

### 2.1 Ethical approval

This study was approved by the Medical Ethics Committee of Wuhan Children's Hospital, affiliated with Huazhong University of Science and Technology (Approval No. 2022R093-F02), and was conducted in accordance with the principles of the Declaration of Helsinki. The ethics committee waived the requirement for individual informed consent due to the retrospective nature of the study, which involved electronic medical records. All data were de-identified and protected by privacy preservation measures.

### 2.2 Data source

Clinical data were extracted for patients clinically diagnosed with IESS at our hospital between January 2018 and December 2023 using Microsoft Excel 2011. All collected data underwent a rigorous evaluation process. For all clinical variables, confirmation was obtained from at least two pediatric neurologists. In cases of disagreement, a senior pediatric neurology specialist was consulted for final adjudication.

### 2.3 Participants

The inclusion criteria for IESS patients in this study were as follows: (1) a diagnosis of IESS according to the criteria established by the International League Against Epilepsy (ILAE) in 2022 (18); (2) a follow-up duration of ≥6 months and ≥2 hospital admissions. Enrolled children were required to have undergone standardized, comprehensive clinical assessments, including cranial magnetic resonance imaging (MRI) and video-EEG monitoring. Exclusion criteria were as follows: (1) incomplete case information, such as unclear medical history or missing crucial ancillary test results; (2) unknown seizure status at the final follow-up or lack of a follow-up EEG record. Any case with missing information was excluded from the study. All findings with uncertain significance regarding IESS etiology or treatment response were subject to final confirmation by a senior pediatric neurology specialist.

**Figure 1:**
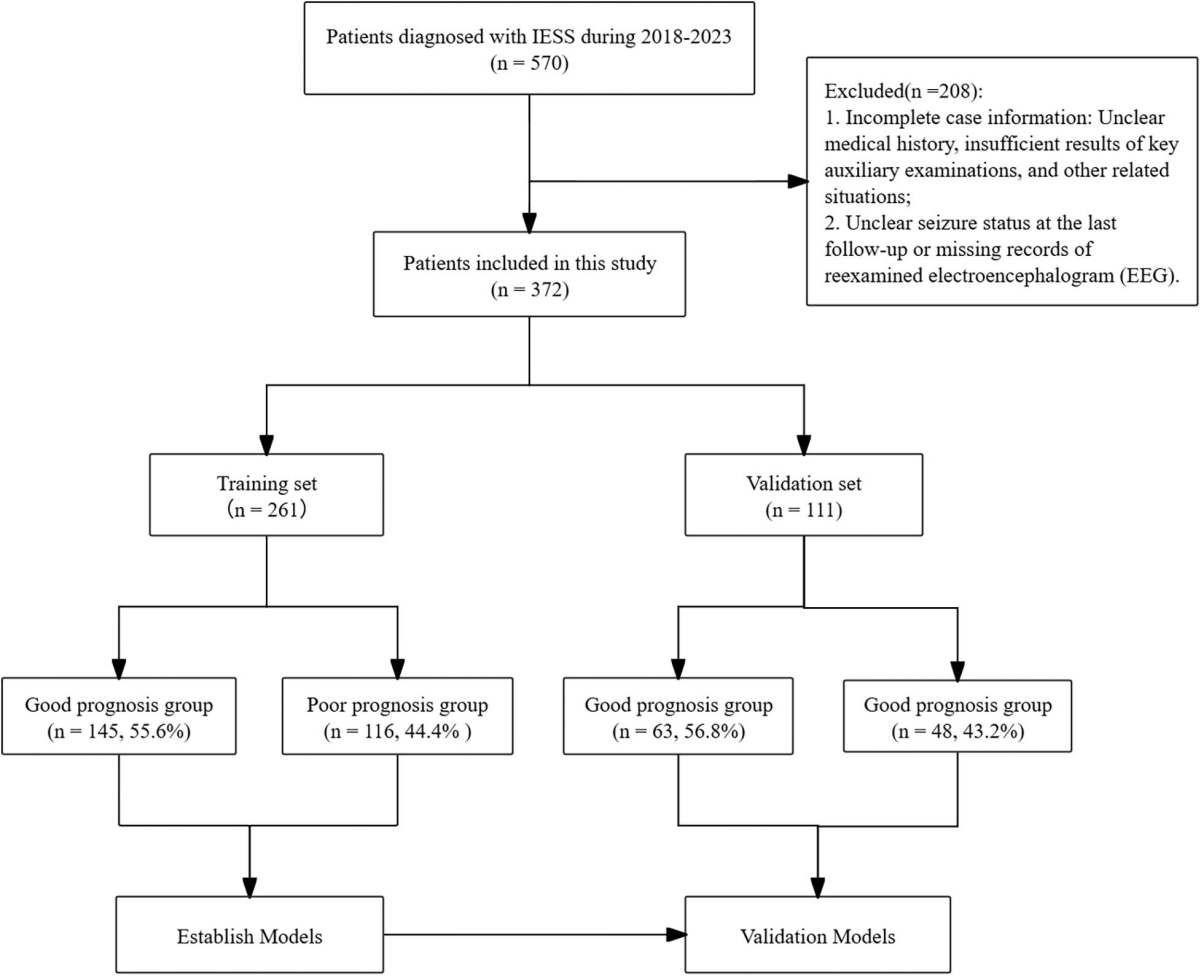
Flowchart fnor selection procedure of patients with IESS.

A total of 570 patients diagnosed with IESS between 2018 and 2023 were initially recruited. After rigorous screening, 208 patients were excluded. Ultimately, 372 patients met the inclusion criteria and were included in the study. These participants were then divided into a training set (*n* = 261, 70%) and a validation set (*n* = 111, 30%), with the validation set serving as an internal cohort for model evaluation (Figure 1).

### 2.4 Outcomes

The primary outcome of this study was the seizure prognosis following treatment for IESS. The criteria for determining seizure outcome were based on the study by Yamada et al. (5). An unfavorable seizure outcome was defined as the presence of either epileptic spasms or a hypsarrhythmic EEG background at the last follow-up. A favorable seizure outcome was defined as the absence of both epileptic spasms and a hypsarrhythmic EEG pattern at the final follow-up assessment.

### 2.5 Data collection and variables

Clinical data were retrospectively collected and included the following variables:

Demographics and History: Sex, age at onset, presence of perinatal abnormalities, and family history of epilepsy.

Disease Course and Characteristics: Time interval from symptom onset to formal diagnosis (diagnostic delay), time interval from symptom onset to treatment initiation (treatment delay), frequency of spasms at onset, and types of seizures present (isolated spasms vs. spasms combined with other seizure types).

Etiology and Examination: Etiology was classified as either unknown or known, with known causes including genetic, structural, and metabolic factors. Data from the initial neurological examination were recorded, along with the presence of developmental delay both at disease onset and at the final follow-up.

Investigative Findings: Results from cranial MRI were categorized as normal/non-specific or abnormal. Specific structural abnormalities were documented, including encephalomalacia, TSC, neuronal migration disorders, brain injury, cerebral atrophy, corpus callosum dysgenesis, and tumors. EEG findings, particularly the presence or absence of hypsarrhythmia, were collected from both the initial and final follow-up assessments.

Treatment and Response: The total number of ASMs used, initial use of first-line ASMs (ACTH, corticosteroids, vigabatrin [VGB]), subsequent initiation of ACTH therapy, and clinical response to both ACTH and ketogenic diet (KD) therapy were evaluated. The use of surgical interventions was also recorded.

Outcome Measures: Data on spasm occurrence and video-EEG report findings from both the initial diagnosis and the final follow-up visit were collected as primary outcome measures.

### 2.6 Model development and validation

Six ML algorithms were used to develop prediction models: Logistic Regression (LR), Random Forest (RF), Support Vector Machine (SVM), Classification and Regression Tree (CART), eXtreme Gradient Boosting (XGBoost), and Light Gradient Boosting Machine (LightGBM).

A standardized data preprocessing pipeline was first applied. Features with missing values exceeding 50% of the total cohort were excluded. For the remaining features, missing numerical values were imputed with the mean value from the training set, while missing categorical values were designated as a separate category, “(Missing)”. All preprocessing parameters, such as imputation values, were strictly derived from the training set and applied consistently to the validation set to ensure proper data handling and prevent data leakage.

Next, systematic feature engineering was performed. Numerical features underwent variance thresholding and removal of highly correlated features to eliminate low-variability or redundant features. Categorical features were encoded using a full-rank one-hot encoding scheme. All features were then standardized, and near-zero variance features were removed, resulting in a refined feature set for subsequent model training.

For model construction and validation, R software (version 4.5.1) and the caret package were used to partition the dataset of 372 patients. The cohort was randomly split into a training set (*n* = 261) and an internal validation set (*n* = 111) at a 7:3 ratio. To minimize sampling bias, stratified random sampling was performed based on the spasm outcome using the caret::createDataPartition function (*p* = 0.7), ensuring the distribution of favorable and unfavorable outcomes in the training and validation sets reflected the original dataset. A fixed random seed (SEED = 1024) was set to ensure reproducibility.

To enhance model robustness, a 5-fold cross-validation strategy was employed. Hyperparameter optimization was performed through stratified cross-validation, aiming to maximize the Area Under the Curve (AUC) on the validation set. The optimal hyperparameters were selected through grid search or random search to ensure a good model fit while mitigating overfitting.

Model performance was evaluated on both the training and independent validation sets. In the training set, out-of-fold (OOF) predictions from cross-validation were used to estimate performance, helping to reduce overfitting. The performance on the validation set was used to assess generalization capability. Evaluation metrics included the Area Under the Receiver Operating Characteristic Curve (AUROC) and its 95% confidence interval, accuracy, precision, recall, specificity, and F1-score. Calibration plots were used to assess the agreement between predicted probabilities and observed outcomes, and Decision Curve Analysis (DCA) was employed to evaluate the clinical net benefit across different probability thresholds. A fixed random seed was set for all analyses to ensure reproducibility.

To further elucidate the model's predictive mechanism, the SHAP (SHapley Additive exPlanations) method was employed to analyze feature contributions. Additionally, to gain deeper insights into the model's decision logic, the structure of a specified decision tree (Tree = 0) from the trained XGBoost model was visualized. The procedure involved extracting key attributes from each node, including the splitting variable, splitting threshold, branching direction, and leaf node output value. These elements were then rendered into a hierarchical binary tree diagram. In the visualization, each internal node partitions the samples based on the rule “whether the variable value ≤ threshold” and is annotated with the corresponding Gain, reflecting its contribution to model performance improvement. Leaf nodes are labeled with their Value, indicating the additive adjustment of that path to the final prediction score. To enhance readability, the display depth was limited to the first four layers from the root node. Given that variables were standardized during model development, negative threshold values in the figure indicate that the variable lies below its mean, rather than representing an actual negative clinical measurement.

### 2.7 Statistical analysis

This study was designed and analyzed following the Transparent Reporting of a Multivariable Prediction Model for Individual Prognosis or Diagnosis (TRIPOD) statement. Due to its retrospective nature, *a priori* sample size calculation was not conducted; instead, the final sample size was determined based on the total number of eligible patients who met the inclusion and exclusion criteria between January 2018 and December 2023.

Categorical variables are presented as numbers and percentages [*n* (%)], with comparisons between groups made using Fisher's exact test or the Chi-square test, as appropriate. Continuous variables were first assessed for normality using the Shapiro–Wilk test. Data that conformed to a normal distribution are expressed as mean ± standard deviation and were compared using the independent samples *t*-test. Non-normally distributed data are expressed as median with interquartile range [*M* (*Q*_1_, *Q*_3_)] and were compared using the Mann–Whitney *U* test. A two-sided significance level (*α*) of 0.05 was set for all statistical tests, with a *P*-value of less than 0.05 considered statistically significant.

In the training set, univariable Cox regression analyses were initially conducted to assess the relationship between clinical features and spasm seizure/EEG prognosis. Variables with a *P*-value less than 0.2 in the univariable analyses were included in a multivariable Cox regression model to identify significant independent prognostic factors, with a *P*-value of less than 0.05 considered significant in the final model. The selection of variables for the multivariable analysis was performed using a backward stepwise selection process based on the likelihood ratio test. All Cox regression analyses were conducted using SPSS software (Version 26.0; IBM Corp), with a two-sided *P*-value <0.05 considered statistically significant.

A variety of machine learning algorithms were employed to develop prognostic prediction models, including Logistic Regression (LR), Random Forest (RF), Support Vector Machine (SVM), Classification and Regression Tree (CART), LightGBM, and XGBoost. All models were implemented using their respective R software packages.

Model predictive performance was evaluated using the Area Under the Receiver Operating Characteristic Curve (AUC). Calibration curves were plotted to assess the agreement between predicted probabilities and actual observations, with the Hosmer-Lemeshow test used to quantify calibration (a *p*-value >0.05 indicating good calibration). Decision Curve Analysis (DCA) was further performed to calculate the net benefit across various probability thresholds, thereby evaluating the clinical utility of the models. These analyses were conducted using the “pROC”, “rms”, and “rmda” R packages (https://github.com/mdbrown/rmda), respectively.

To enhance model interpretability, feature importance based on the Gain metric was computed for the best-performing XGBoost model. Additionally, the first decision tree within the ensemble was extracted and visualized to illustrate node-splitting variables, threshold values, and leaf node outputs. This visualization was generated using the “DiagrammeR” package, with display depth limited to four layers for improved readability. All statistical analyses were performed in the R 4.3.0 environment (https://www.r-project.org), utilizing primarily the caret, xgboost, pROC, and DiagrammeR packages. (https://www.r-project.org).

## 3 Results

### 3.1 Demographic and clinical characteristics

Figure 1 depicts the patient screening process, with 372 participants ultimately included in the study. Based on the outcome criteria at the final follow-up (median follow-up time: 18 months), 208 children (56%) were classified as having a favorable outcome, defined by the absence of spasms and resolution of hypsarrhythmia on EEG. In contrast, 164 children (44%) had an unfavorable outcome, characterized by the persistence of either spasms or a hypsarrhythmic EEG pattern. The demographic and clinical characteristics of all participants are summarized in Table 1.

**Table 1:** Comparison of demographic characteristics between the training set and the validation set.

Characteristics	Overall, *N* = 372^1^	Training cohort, *N* = 261^1^	Internal testing cohort, *N* = 111^1^	*P*
Gender, *n* (%)				0.757^3^
Female	143 (38.4%)	99 (37.9%)	44 (39.6%)	
Male	229 (61.6%)	162 (62.1%)	67 (60.4%)	
Duration of follow-up (day)				0.482^2^
Median (IQR)	1,149 (652.5, 1,926.75)	1,145 (679.0, 1,915.0)	1,181 (617.0, 1,927.0)	
Age at onset of IESS (months)				0.833^2^
Median (IQR)	5.0 (3.0, 7.0)	5.0 (3.0, 7.0)	5.0 (3.0, 7.0)	
Diagnosis lag (days)				0.457^2^
Median (IQR)	31.0 (13.0, 67.5)	31.0 (13.0, 71.5)	30.0 (13.0, 61.0)	
Treatment lag (days)				0.505^2^
Median (IQR)	33.5 (15.0, 72.0)	33.0 (15.0, 86.0)	34.0 (15.0, 68.0)	
Number of spasms at diagnosis				0.851^2^
Median (IQR)	5.0 (3.0–8.0)	5.0 (3.0–8.0)	5.0 (3.0–8.0)	
Etiology, *n* (%)				0.756^3^
Unknown	219 (58.9%)	155 (59.4%)	64 (57.7%)	
Known	153 (41.1%)	106 (40.6%)	47 (42.3%)	
Genetic	80 (21.5%)	59 (22.6%)	21 (18.9%)	0.428^3^
Structural	92 (24.7%)	65 (24.9%)	27 (24.3%)	0.906^3^
Metabolic	8 (2.2%)	5 (1.9%)	3 (2.7%)	0.930^3^
Perinatal abnormalities, *n* (%)				0.658^3^
Yes	108 (29.0%)	74 (28.4%)	34 (30.6%)	
No	264 (71.0%)	187 (71.6%)	77 (69.4%)	
Family history, *n* (%)				0.989^3^
Yes	15 (4.0%)	10 (3.8%)	5 (4.5%)	
No	357 (96.0%)	251 (96.2%)	106 (95.5%)	
Developmental delay at onset of IESS, *n* (%)				0.377^3^
Yes	256 (68.2%)	176 (67.4%)	80 (72.1%)	
No	116 (31.2%)	85 (32.6%)	31 (27.9%)	
Developmental delay at the last follow-up, *n* (%)				0.575^3^
Yes	337 (90.6%)	235 (90.0%)	102 (91.9%)	
No	35 (9.4%)	26 (10.0%)	9 (8.1%)	
Neurologic examination at onset of IESS, *n* (%)				0.596^3^
Yes	97 (26.1%)	66 (25.3%)	31 (27.9%)	
No	275 (73.9%)	195 (74.7%)	80 (72.1%)	
Seizure types, *n* (%)				0.853^3^
Epileptic spasms only	269 (72.3%)	188 (72.0%)	81 (73.0%)	
Spasm with other seizure types	103 (27.7%)	73 (28.0%)	30 (27.0%)	
Hypsarrhythmia, *n* (%)				0.503^3^
Typical/Atypical Hypsarrhythmia	259 (69.6%)	179 (68.6%)	80 (72.1%)	
No Hypsarrhythmia	113 (30.4%)	82 (31.4%)	31 (27.9%)	
MRI findings, *n* (%)				0.886^3^
Normal	246 (66.1%)	172 (65.9%)	74 (66.7%)	
Abnormal	126 (33.9%)	89 (34.1%)	37 (33.3%)	
MRI abnormalities-types				
Encephalomalacia	65 (17.5%)	47 (18.0%)	18 (16.2%)	0.677^3^
Coexisting TSC	17 (4.6%)	12 (4.6%)	5 (4.5%)	0.969^3^
Neuronal Migration Disorder	19 (5.1%)	13 (5.0%)	6 (5.4%)	0.865^3^
Brain injury	14 (3.8%)	6 (2.3%)	8 (7.2%)	0.048^3^
Brain Atrophy	12 (3.2%)	7 (2.7%)	5 (4.5%)	0.555^3^
Dysgenesis of corpus callosum	6 (1.6%)	6 (2.3%)	0	0.246^3^
Tumor	4 (1.1%)	1 (0.4%)	3 (2.7%)	0.151^3^
Initiating treatment with ACTH, *n* (%)				0.789^3^
Yes	251 (67.5%)	175 (67.0%)	76 (68.5%)	
No	121 (32.5%)	86 (33.0%)	35 (31.5%)	
Response to ACTH therapy at day 14, *n* (%)				0.961^3^
Effective	131 (35.2%)	91 (34.9%)	40 (36.0%)	
Ineffective	120 (32.3%)	84 (32.2%)	36 (32.4%)	
Number of total ASMs, *n* (%)				0.402^2^
Median (IQR)	4.0 (2.0 , 5.0)	4.0 (2.0, 5.0)	4.0 (2.0, 5.0)	
Use of first-line ASMs, *n* (%)				0.755^3^
Yes	220 (59.1%)	153 (58.6%)	67 (60.4%)	
No	152 (40.9%)	108 (41.4%)	44 (39.6%)	
Initiating treatment with KD, *n* (%)				0.205^3^
Yes	118 (31.7%)	88 (33.7%)	30 (27.0%)	
No	254 (68.3%)	173 (66.3%)	81 (73.0%)	
Response to KD therapy, *n* (%)				0.443^3^
Effective	38 (10.2%)	28 (10.7%)	10 (9.0%)	
Ineffective	80 (21.5%)	60 (23.0%)	20 (18.0%)	
Surgery, *n* (%)				0.883^3^
Yes	29 (7.8%)	20 (7.7%)	9 (8.1%)	
No	343 (92.2%)	241 (92.3%)	102 (91.9%)	

IESS, infantile epileptic spasm syndrome; TSC, Tuberous Sclerosis Complex; ACTH, adrenocorticotropic hormone; KD, ketogenic diet; ASMs, Antiseizure medications.

^1^ *n* (%).

^2^ Wilcoxon rank sum test/Mann–Whitney U test.

^3^ Pearson's Chi-squared test.

The median age at onset was 5.0 months (range: 3–7 months), with a male predominance (61.6%). The median time to diagnosis was 31 days, and the median time to treatment initiation was 33.5 days, indicating a slight treatment delay of approximately 2 days post-diagnosis for most children. Regarding clinical features, 103 patients (27.7%) experienced other seizure types in addition to epileptic spasms, including focal seizures, generalized tonic seizures, and myoclonic seizures. Interictal EEG monitoring revealed that 259 children (69.6%) exhibited typical or atypical hypsarrhythmia, while 113 (30.4%) did not. The median frequency of daily spasms prior to diagnosis was 5.0 episodes (range: 3–8). At the time of initial diagnosis, 86.0% of patients had normal neurological examinations, while abnormal signs were observed in 52 children (14.0%), primarily hypertonia/hypotonia (86.5%), diminished patellar tendon reflexes (7.7%), and distinctive facial features (5.8%). Additionally, 256 children (68.8%) were diagnosed with developmental delay at presentation, affecting motor, language, and cognitive domains, as confirmed by assessments such as the Gesell Developmental Schedules. Notably, by the last follow-up, 96.0% of children continued to exhibit developmental delay, regardless of spasm control.

Regarding birth history, 71.0% of the children had no documented perinatal abnormalities (such as asphyxia, hypoxia, or infection), and 96.0% had no family history of epilepsy. Etiological analysis revealed that the cause remained unknown in 58.9% of patients, while 41.1% had identified etiologies, including genetic (21.5%), structural (24.7%), and metabolic (2.2%) factors. MRI results were abnormal in 127 children (34.1%). Primary findings included encephalomalacia (17.5%), TSC (4.8%), neuronal migration disorders (5.1%), brain injury (3.8%), cerebral atrophy (3.5%), and space-occupying tumors (1.6%), among others.

Regarding treatment, 59.1% of the children received first-line ASMs (such as ACTH, methylprednisolone, or VGB) as initial therapy. The median number of ASMs used per child was 4. A total of 251 children (67.5%) underwent ACTH therapy, with 52.2% (*n* = 131) showing a favorable response, defined as the complete cessation of spasms within 14 days of treatment. Additionally, 118 children (31.7%) received KD therapy, with 32.2% (*n* = 38) demonstrating a significant reduction in seizure frequency. Surgical interventions, including vagus nerve stimulation or epileptogenic focus resection, were performed on 29 patients (7.8%).

All enrolled children (*n* = 372) were randomly divided into a training set (*n* = 261) and an internal validation set (*n* = 111) at a 7:3 ratio using stratified random sampling. No statistically significant differences in baseline characteristics were observed between the two sets (all *p* > 0.05), confirming their comparability (Table 1). Within the training set, patients were classified into a favorable outcome group (*n* = 145) and an unfavorable outcome group (*n* = 116) based on their status at the last follow-up. Subsequent univariable analysis identified several features with significant differences between the outcome groups (all *p* < 0.05), including etiology, structural etiology, perinatal history, presence of developmental delay, seizure types, MRI findings, response to ACTH therapy, use of KD, and response to KD therapy (Supplementary Table S1). Categorical variables are presented as numbers and percentages, with group comparisons performed using the Chi-square test or Fisher's exact test, as appropriate.

### 3.2 Identification of prognostic factors via logistic regression analysis

Univariable Cox regression analysis within the training set identified several factors significantly associated with seizure outcomes in IESS patients (*P* < 0.2 for inclusion). These included seizure frequency prior to diagnosis, etiology (particularly structural etiology), presence of developmental delay after onset, seizure types (isolated spasms vs. combined types), abnormal MRI findings, response to ACTH therapy, total number of ASMs used, and response to the KD (Supplementary Table S2). Multivariable Cox regression analysis, incorporating these variables, revealed that TSC and neuronal migration disorders were independent risk factors for unfavorable seizure outcomes in children with IESS (Hazard Ratio >1, *P* < 0.05; Table 2).

**Table 2:** Multivariable Cox regression analysis of patients with IESS in the training cohort.

Characteristics	OR	95% CI	*P*
Treatment lag	0.999	0.998–1.001	0.392
Number of spasms at diagnosis	1.007	0.951–1.066	0.815
Genetic	0.863	0.374–1.993	0.730
Metabolic	1.805	0.509–6.396	0.360
Genetic	1.256	0.541–2.920	0.596
Perinatal abnormalities	1.485	0.965–2.287	0.072
Family history	0.619	0.183–2.095	0.441
Developmental delay at onset of IESS	1.467	0.919–2.342	0.108
Developmental delay at the last follow-up	0.958	0.396–2.320	0.924
Seizure types	1.372	0.902–2.089	0.140
Encephalomalacia	0.929	0.387–2.231	0.870
Coexisting TSC	2.729	1.123–6.630	0.027*
Neuronal Migration Disorder	3.948	1.397–11.156	0.010*
Initiating treatment with ACTH	801.778	0.239–2.325	0.0910
Response to ACTH therapy at day 14	1.352	0.826–2.213	0.230
Number of total ASMs	1.084	0.993–1.183	0.073
Use of first-line ASMs	1.419	0.945–2.129	0.091
Initiating treatment with KD	0.846	0.433–1.653	0.624
Response to KD therapy	1.413	0.709–2.817	0.326

* *p* < 0.05.

### 3.3 Model performance

#### 3.3.1 Model development and comparative performance

Six ML algorithms were employed to develop predictive models for seizure outcomes in children with IESS. Model performance was evaluated in both the training and validation sets using AUC, Accuracy, Precision, Specificity, Recall, and F1-Score, with detailed results presented in Table 3 and Supplementary Table S3.

**Table 3:** Performance metrics for six models in validation dataset

Model	AUROC	Accuracy	Precision	Recall	Specificity	F1-score
LR	0.798 [0.696–0.901]	0.743	0.76	0.594	0.857	0.667
RF	0.710 [0.589–0.831]	0.649	0.6	0.562	0.714	0.581
SVM	0.788 [0.683–0.893]	0.716	0.739	0.531	0.857	0.618
CART	0.717 [0.611–0.822]	0.716	0.657	0.719	0.714	0.687
XGB	0.812 [0.714–0.910]	0.757	0.719	0.719	0.786	0.719
GBM	0.784 [0.677–0.891]	0.757	0.706	0.75	0.762	0.727

AUROC, area under the receiver operating characteristic curve; LR, logistic regression; RF, random forest; SVM, support vector machine; CART, classification and regression tree; XGB, eXtreme gradient boosting; LGB, light gradient boosting machine.

In the training set, the XGBoost model achieved the highest AUC (0.827, 95% CI: 0.781–0.874). The performance of the other models, in descending order of AUC, was as follows: LightGBM (AUC: 0.815, 95% CI: 0.767–0.863), LR (AUC: 0.815, 95% CI: 0.766–0.864), RF (AUC: 0.808, 95% CI: 0.759–0.857), SVM (AUC: 0.792, 95% CI: 0.740–0.843), and CART (AUC: 0.770, 95% CI: 0.715–0.824). The XGBoost model also demonstrated an accuracy of 0.748 and an F1-Score of 0.686. In contrast, the CART model had the lowest AUC (0.770), while the SVM model recorded the lowest F1-Score (0.639). These performance metrics were derived from the OOF predictions obtained via cross-validation to reduce overfitting and provide a more robust estimate of the models’ generalization performance.

In the internal validation set, the XGBoost model continued to demonstrate superior predictive performance, achieving the highest AUC of 0.812 (95% CI: 0.714–0.910). Its discriminative ability was complemented by an accuracy of 0.757 and an F1-Score of 0.719. The LR model showed a comparable AUC of 0.798 (95% CI: 0.696–0.901), but its F1-Score was lower at 0.667. The RF model exhibited the weakest performance in the validation set, with the lowest recorded AUC (0.710) and F1-Score (0.581), indicating relatively weaker generalization capability.

In summary, the XGBoost model demonstrated superior discriminative ability and maintained stable performance across both the training and internal validation sets, supporting its selection as the optimal predictive model for this study.

#### 3.3.2 Model validation

A comprehensive evaluation of the model was conducted using ROC curves, calibration curves, and DCA.

**Figure 2:**
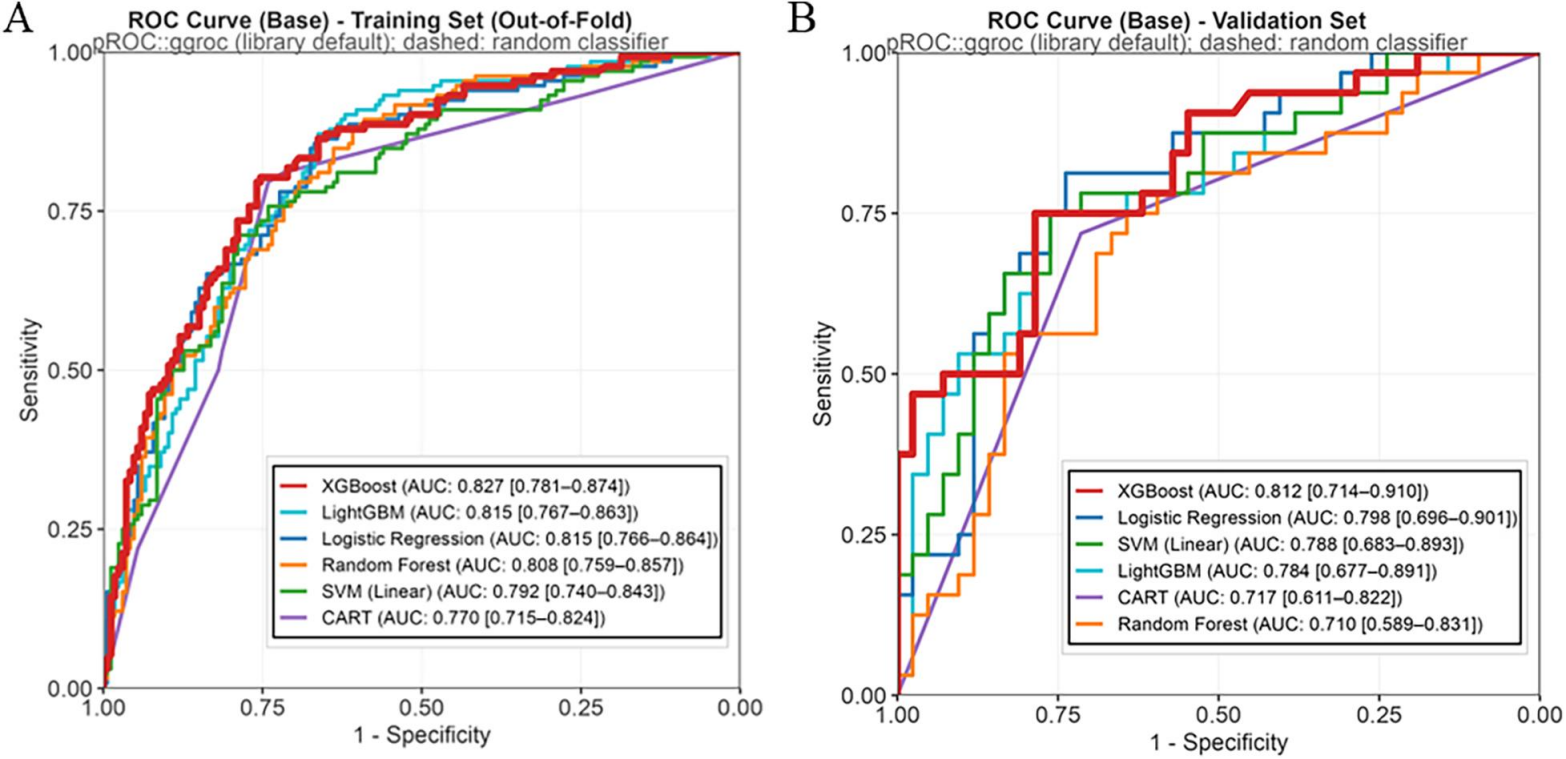
ROC of the machine learning models in the training dataset **(A)** and validation dataset **(B)**.

The ROC curves for both the training and validation sets (Figures 2A,B, respectively) revealed that the XGBoost model achieved the highest AUC values in both cohorts (0.827 in the training set and 0.812 in the validation set). These consistent results demonstrate the model's optimal classification performance and robust generalization capabilities.

**Figure 3:**
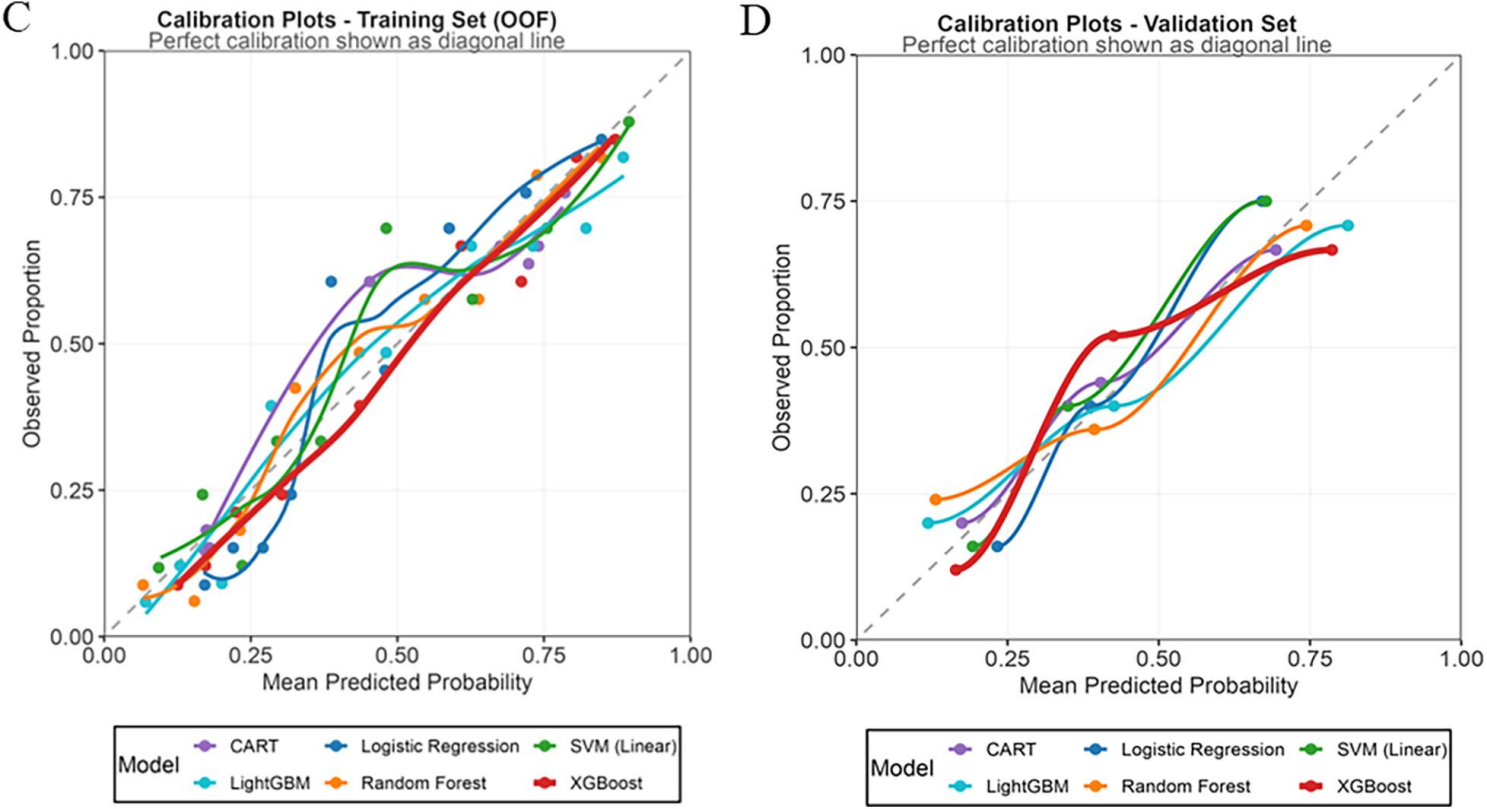
Calibration plot of the machine learning models in the training dataset **(C)** and validation dataset **(D)**.

The calibration curves (Figures 3C,D) indicated excellent calibration performance for both the LightGBM and XGBoost models in the training set, with non-significant HL test p-values of 0.81 and 0.72, respectively. In the validation set, LightGBM demonstrated the best calibration, with its curve most closely aligning with the ideal diagonal (HL test *p* = 0.89). The XGBoost model also exhibited favorable calibration, particularly in the intermediate to high probability range (0.4–0.8), supported by an HL test *p*-value of 0.65. Thus, LightGBM was identified as the best-calibrated model, followed closely by the XGBoost model.

**Figure 4:**
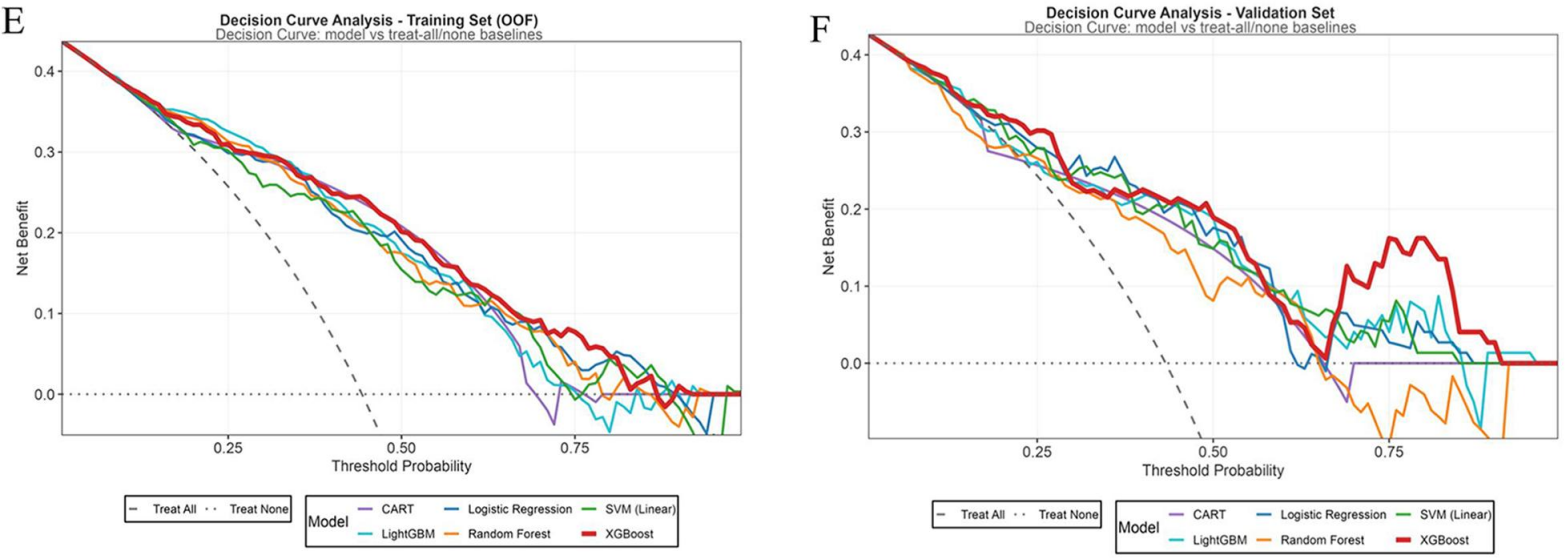
Decision curve analysis of the machine learning models in the training dataset **(E)** and validation dataset **(F)**.

The DCA curves (Figures 4E,F) illustrated that in the training set, the XGBoost model provided a higher net benefit across most threshold probabilities, offering superior decision utility within clinically relevant ranges. Since persistent spasms in children with IESS can severely affect neurodevelopment, clinical practice typically favors lower decision thresholds to facilitate early identification of patients at risk for unfavorable outcomes. In the validation set, the XGBoost model maintained a stable and comparatively high net benefit within the threshold probability range of 0.2–0.5, indicating its strong clinical applicability. Therefore, XGBoost was established as the model with the optimal decision-analytic performance.

In summary, based on the comprehensive analysis of ROC, calibration, and decision curves, the XGBoost model demonstrates outstanding performance in discriminative ability, calibration stability, and clinical decision support, making it the recommended model for predicting seizure outcomes in children with IESS.

### 3.4 Model interpretability

**Figure 5:**
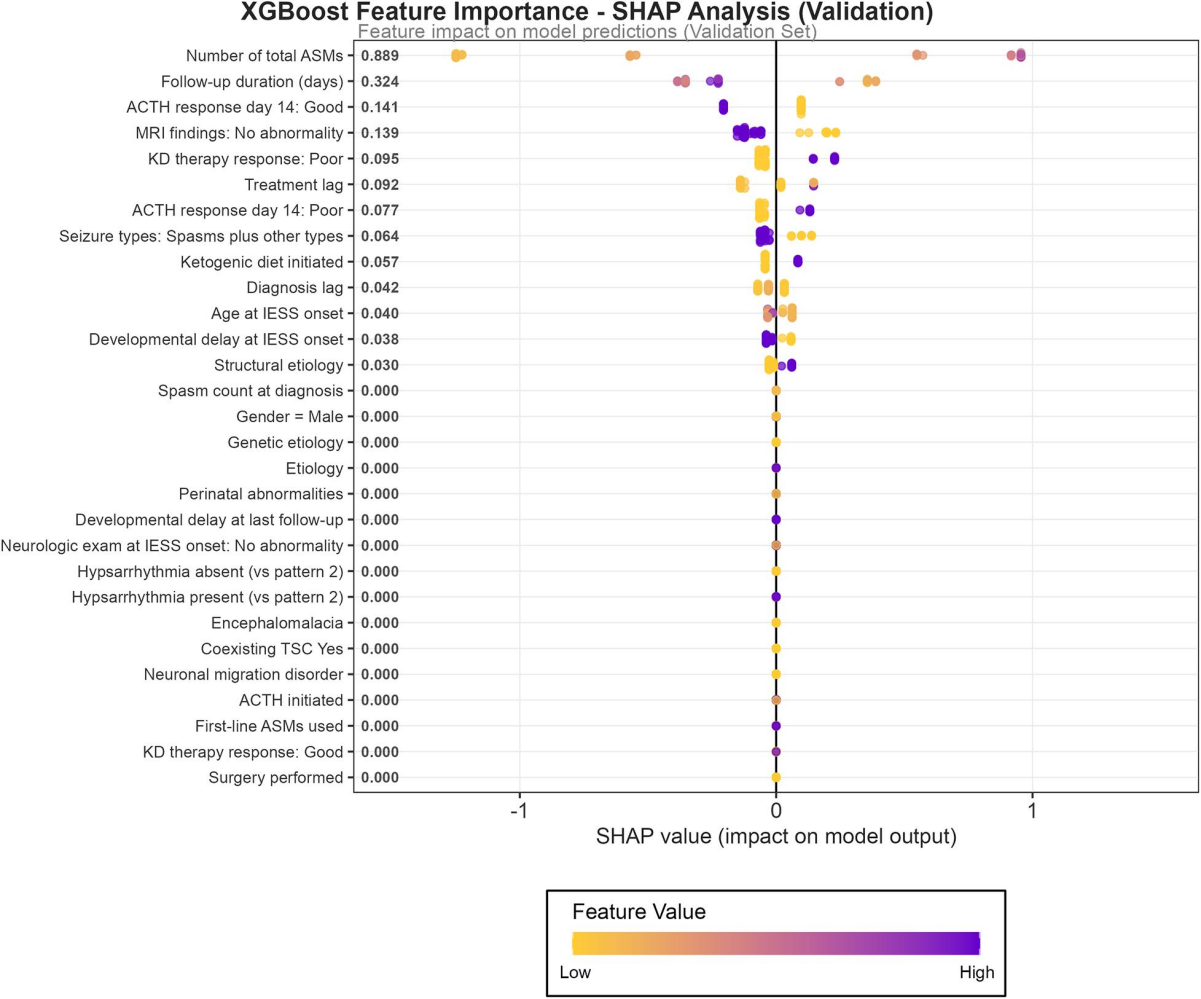
Feature importance in terms of the XGBoost machine learning model.

To enhance the interpretability of the optimal predictive model, the SHAP method was applied to identify the key features influencing the predictions of the XGBoost model. The summary plot (Figure 5) reveals that the total number of ASMs used was the most influential factor, exerting the strongest impact on the model's output. This was followed in importance by follow-up duration, initiation of the KD, time to treatment initiation, and MRI findings. Together, these five variables formed the core set of predictors, offering valuable insights into the model's decision-making process and highlighting potential areas for clinical intervention.

### 3.5 Decision tree visualization

**Figure 6:**
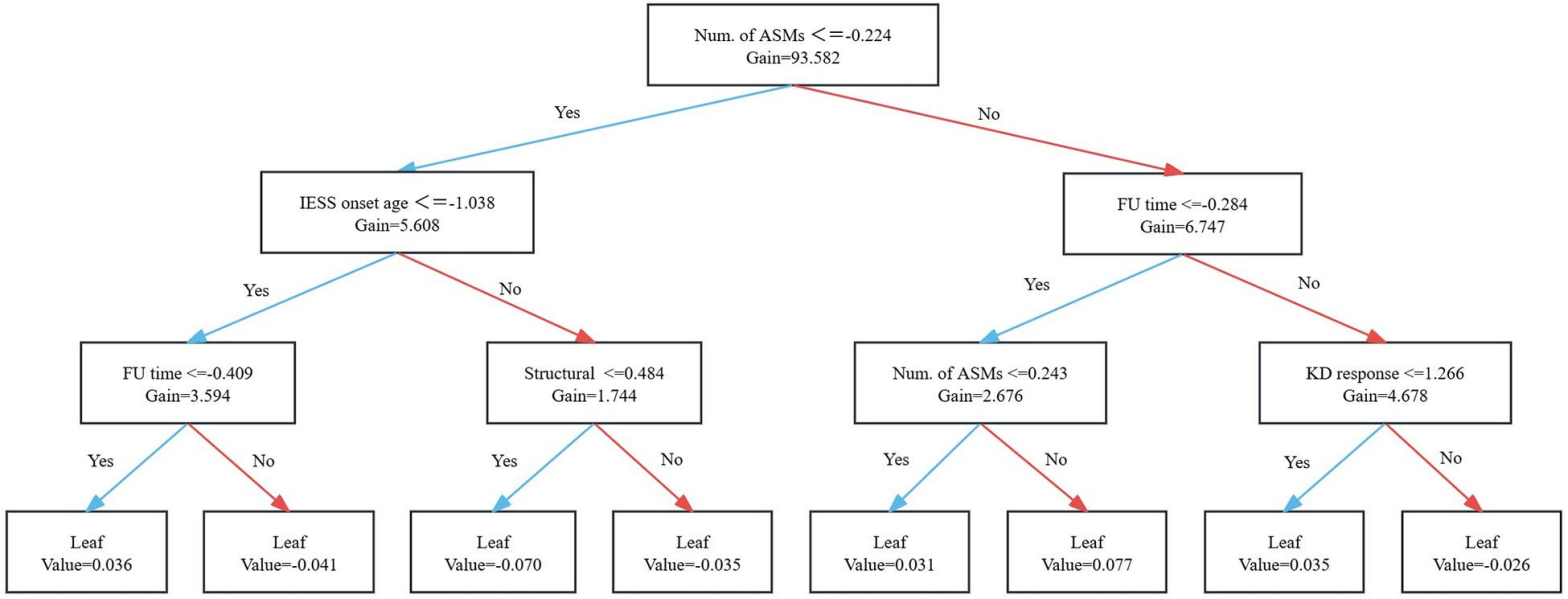
Visualization of a single decision tree in the XGBoost model: feature paths and predictive outputs.

Figure 6 visualizes the hierarchical structure of a single decision tree from the XGBoost ensemble, demonstrating a “core-feature-led, progressive refinement” approach. This method systematically partitions the complex data space of IESS patients into distinct subgroups with discernible prognostic differences. The tree starts with the number of ASMs as the root node (split threshold <−0.224, Gain = 93.582), representing the most significant initial partition of the patient cohort into two primary subgroups. This root split establishes the fundamental prognostic distinction between patients who require fewer versus more ASMs, confirming this feature as the most critical predictor.

Subsequent splits integrate secondary clinically relevant features to progressively reduce within-group prognostic heterogeneity. In the subgroup with fewer ASMs, further branches occur based on Age at Onset (<−1.038), Follow-up Time, and the presence of Structural Abnormalities on neuroimaging. This process results in four terminal subgroups defined by unique combinations of these features, yielding SHAP prediction values of 0.036, −0.041, −0.070, and −0.035, respectively.

In contrast, within the subgroup requiring more ASMs, partitioning occurs sequentially based on Follow-up Time (<−0.284), a secondary split on the number of ASMs, and finally response to KD therapy. This generates another four terminal subgroups, with prediction values of 0.031, 0.077, 0.035, and −0.026.

This schematic provides an intuitive representation of how a single decision tree stratifies the sample space into prognostic subgroups based on feature thresholds. The final XGBoost model prediction for any given patient is derived by aggregating the outputs from multiple such trees. The visualization clearly delineates the hierarchical prediction pathways shaped by different features and their interactions, offering an interpretable framework to understand the relative importance of clinical variables and their differential effects across patient subgroups.

## 4 Discussion

IESS, a developmental epileptic encephalopathy, typically presents in infancy and early childhood, with most affected children experiencing onset within the first year of life, and only rare cases emerging after two years of age (2, 19). Despite its relatively low incidence, IESS is linked to poor outcomes and significant long-term sequelae, such as reduced intellectual quotients, an increased risk of autism spectrum disorders, and diminished quality of life, making it a critical focus of clinical research in recent years (4). Previous studies have primarily focused on etiological classification and univariable prognostic analyses of IESS, with a notable lack of systematic, generalizable predictive models. Thus, developing an accurate predictive tool is essential for identifying factors that significantly influence IESS prognosis, enabling early risk stratification, and facilitating the development of personalized treatment strategies.

In recent years, ML techniques, particularly the XGBoost model, have been widely explored in the medical field (20), including applications for predicting seizure occurrence and recurrence in epilepsy (21, 22). However, research on the use of XGBoost for prognostic prediction in epilepsy remains limited and underexplored, despite its potential (23). The present study aimed to integrate multiple ML techniques to develop a predictive model for seizure outcomes in IESS based on clinical and EEG data. By enabling early identification of key clinical features and assisting in the evaluation of etiology and treatment strategies, this model provides a valuable tool for prognostic assessment in children with IESS, offering data-driven support for clinical analysis and decision-making.

In this study, patients were divided into a training set and an internal validation set. Initially, univariable Cox regression analysis within the training set was used to identify risk factors associated with seizure outcomes in IESS patients. The results indicated several factors linked to an unfavorable seizure prognosis, including seizure frequency prior to diagnosis, etiology, structural etiology, presence of developmental delay after onset, seizure types, abnormal MRI findings, response to ACTH therapy, total number of ASMs used, and response to KD therapy. These factors align with findings in previous studies, although some failed to reach statistical significance in the current analysis, likely due to factors such as sample size or study design limitations. The etiology of IESS is diverse, generally categorized into symptomatic (approximately two-thirds) and cryptogenic (approximately one-third) types. Recent ILAE classifications further refine this into metabolic, genetic, infectious/immune, and unknown groups (24, 25). Literature suggests that over 25 inborn errors of metabolism are recognized as causative factors for IESS, including common disorders such as phenylketonuria and methylmalonic acidemia (26). With advancements in genetic testing, the role of genetic factors in IESS pathogenesis has gained increasing recognition. For instance, the ARX (Aristaless) gene, essential for the development of GABAergic interneurons, if dysfunctional, can cause neuronal migration and functional impairments, leading to seizures (27). Similarly, mutations in the Cyclin-Dependent Kinase-Like 5 (CDKL5) gene, which is involved in neuronal maturation and synaptogenesis, can result in severe neurodevelopmental delay and seizures, manifesting as West syndrome among other phenotypes (28). Additionally, hypoxic-ischemic brain injury during the neonatal period, particularly prevalent in developing countries, remains a major etiology of IESS (29, 30). Notably, experimental findings from Merve et al. suggested that patients with structural etiologies tend to have more favorable outcomes compared to those with metabolic or genetic causes (31). Therefore, considering IESS etiology in predictive modeling is both rational and critical. Pre-onset developmental delay has long been recognized as a significant prognostic factor in IESS. A study by Raili Riikonen et al. found that children with significant developmental delay prior to onset generally had poorer prognoses and developmental outcomes, consistent with our findings (4). Consequently, closer attention to prognosis and timely adjustments to treatment strategies are recommended for patients with difficult-to-identify etiologies or clear developmental delay at onset.

Children with IESS exhibit heterogeneity in seizure semiology, and this phenotypic variability plays a significant role in differences in patient prognosis. For example, Cuccurullo et al. reported that among IESS cases linked to pathogenic variants in the DYNC1H1 gene (which encodes cytoplasmic dynein 1 heavy chain 1), 60% of patients developed pharmacoresistant epilepsy, while 25% experienced progression of their epileptic phenotype to Lennox-Gastaut syndrome (LGS). This progression was marked by the emergence of tonic, clonic, and atonic seizures, along with abnormal EEG patterns, all indicating a generally poor prognosis (32). This finding suggests that the duration and evolutionary trajectory of seizures in IESS may significantly affect long-term outcomes. Supporting this, a 2023 study by Sharawat et al. found that children with IESS secondary to nutritional vitamin B12 deficiency typically exhibited fewer spasm clusters and fewer spasms per cluster, correlating with more favorable prognoses (33). This may suggest that lower seizure frequency and reduced severity lead to less cerebral insult, thereby fostering conditions that are more conducive to neurodevelopmental recovery.

In this study, the number of ASMs used correlated with unfavorable seizure outcomes in univariable analysis but did not reach statistical significance in multivariable analysis, potentially due to the relatively small sample size. Previous studies have reported inconsistent findings regarding the relationship between the number of ASMs and seizure prognosis. On one hand, the use of multiple ASMs may reflect greater disease severity, while on the other hand, drug interactions could impact seizure control. For example, a 2025 study demonstrated that combining prednisolone and VGB, a GABA analogue, was more effective in promoting seizure remission than monotherapy with either drug alone (34). Thus, the significance of ASM number warrants further investigation. Currently, oral prednisolone or intramuscular ACTH are widely accepted as first-line treatments for IESS (35). Additionally, 70% of children treated with prednisolone and 76% treated with ACTH achieve complete cessation of spasms within 14 days (36). However, patients with developmental delay prior to spasm onset may have a poorer response to ACTH therapy, and those with specific cranial MRI abnormalities may be at higher risk of relapse (37). For children who do not respond to first-line therapies, second-line treatments, such as the KD, may be considered. A meta-analysis of non-randomized clinical trials indicated that KD can reduce spasm frequency by more than 50% (38). However, research on KD for IESS remains limited, with many studies involving small sample sizes. Therefore, larger-scale randomized controlled trials are needed to validate the efficacy of the KD (39). Consistent with previous findings (37), our study confirmed that a favorable response to ACTH therapy is associated with better outcomes. Accordingly, Arai et al. recommended that if epileptic spasms do not respond to initial ACTH treatment, clinicians should promptly initiate subsequent therapies, including a second course of ACTH, VGB, surgical intervention, or dietary treatments (6). Furthermore, response to KD is closely linked to prognosis in IESS patients, and the sequence of KD and ACTH administration appears clinically relevant. For instance, a 2025 study by Dressler et al. reported that patients treated with KD followed by ACTH showed better outcomes than those treated with the reverse sequence (ACTH followed by KD) (40). In conclusion, developing individualized medication plans and making timely adjustments are crucial for children with IESS. A comprehensive understanding of the patient's medication history is essential for guiding clinical decision-making and optimizing treatment strategies.

The multivariable Cox regression analysis in this study identified two independent risk factors for an unfavorable prognosis in IESS: TSC and neuronal migration disorders—both recognized as significant neurodevelopmental structural abnormalities in IESS. TSC is a rare genetic disorder affecting multiple organ systems, with a prevalence ranging from 1 in 6,000 to 1 in 10,000 individuals (41). Over 85% of TSC cases are linked to pathogenic variants in the TSC1 or TSC2 genes, leading to inactivation of the encoded proteins and subsequent hyperactivation of the mTOR signaling pathway (42). Epilepsy is one of the most common neurological manifestations of TSC, affecting approximately 90% of patients, with 38%–50% developing drug-resistant epilepsy (42, 43). As a significant risk factor for IESS, nearly 40% of TSC patients with epilepsy present with IESS (44). In addition to epilepsy, TSC is associated with various neurological issues, including cortical tubers, subependymal nodules, and subependymal giant cell astrocytomas, which can lead to cerebral malformations such as cortical tubers (45, 46). The development of the human cerebral cortex is a complex process, with radial migration of excitatory neurons playing a critical role (47). Disruptions in neuronal migration, preventing excitatory precursors from migrating properly or causing them to settle in aberrant locations, can lead to severe congenital brain malformations, such as lissencephaly, pachygyria (characterized by abnormally large and thick gyri), polymicrogyria (excessively small and numerous gyri), and cobblestone malformations (48, 49). While no study has definitively established a direct causal link between neuronal migration disorders and IESS, epilepsy is commonly observed in patients with malformations of cortical development (MCD), even when lesions are not located in anatomically critical regions (50).

MRI is the first-line method for determining the etiology of IESS, with over half of affected children showing detectable abnormalities on imaging. MRI plays a pivotal role in guiding diagnostic and therapeutic decisions (18). For instance, lesions in structures like the basal ganglia, thalamus, hypothalamus, and brainstem (e.g., tumors) can disrupt cortical-subcortical networks and trigger IESS. MRI allows for precise anatomical localization of the underlying etiology (51). Notably, the two independent risk factors for an unfavorable prognosis identified in our study—TSC and neuronal migration disorders—can both be diagnosed and characterized via MRI. In fact, disorders caused by neuronal migration disturbances can even be detected prenatally through fetal MRI (52). Our findings align with and reinforce existing literature, highlighting the critical role of neuroimaging in the prognostic evaluation of IESS. Consequently, performing MRI early in the diagnostic process and conducting follow-up scans when indicated is essential for accurately assessing long-term outcomes in children with IESS.

In this study, multiple ML models, including RF, SVM, XGBoost, and LightGBM, were utilized to predict seizure outcomes in children with IESS. The results indicated that the XGBoost model outperformed the others, achieving AUC values of 0.827 in the training set and 0.812 in the validation set, alongside high accuracy, sensitivity, and specificity. This superior performance can be attributed to XGBoost's core mechanism of iteratively optimizing decision trees through gradient boosting, which allows the model to effectively capture complex nonlinear relationships. Thus, XGBoost is particularly well-suited to handle multimodal clinical data (53). Supporting this, Yossofzai et al. developed and validated an XGBoost model to predict seizure outcomes following pediatric epilepsy surgery, demonstrating its high predictive accuracy with epilepsy-related clinical data and strong performance across multicenter datasets (54). XGBoost is highly adaptable in both large and medium-sized sample studies, effectively mitigating overfitting through its built-in regularization techniques (55, 56). Thus, the XGBoost model proves particularly effective in processing the multidimensional clinical data of IESS patients, enabling more accurate identification of patient risk stratification and providing more precise prognostic predictions.

Despite the strong accuracy and predictive performance of the ML model, its “black-box” nature remains a significant barrier to clinical adoption. To address this issue of interpretability, the SHAP method was used to analyze feature importance within the XGBoost model (57). The results identified several key features—including the number of ASMs, follow-up duration, presence of MRI abnormalities, and response to ACTH therapy—that contributed most significantly to the model's predictions. Within the model, each terminal branch corresponds to a distinct patient subgroup, defined by a specific combination of clinical features, with the output value representing the estimated prognosis for that subgroup. Through hierarchical splitting, the model incorporates five key variables—“number of ASMs, age at onset, follow-up time, structural abnormalities, and response to KD”—to reveal their conditional predictive relationships. For example, within the branch representing the lowest medication exposure, age at onset becomes the secondary dominant factor determining prognosis. Conversely, in the high medication exposure pathway, follow-up duration and structural abnormalities refine the predictions. Notably, the subgroup characterized by “high medication load+mild structural abnormalities + long follow-up” exhibited a lower risk than the “high medication load + short follow-up” subgroup, suggesting a significant interaction between structural damage and treatment duration. Ultimately, the eight terminal nodes define a continuous gradient of risk from low to high. This stratification allows clinicians to quickly assess a child's prognostic level and make informed decisions about whether to maintain the current treatment regimen, expedite second-line therapy, or evaluate the timing for surgical intervention or KD. In this way, the model facilitates individualized patient management. This interpretable framework, illustrated in Figure 6, bridges the gap between complex model predictions and clinically actionable insights.

Despite the significant findings, this study has several limitations (23, 58). First, its retrospective design and reliance on electronic medical records render it susceptible to selection and information bias, as variability in clinical documentation practices may have compromised data accuracy and consistency. Second, as a single-center study, the findings may not fully account for the heterogeneity of patient populations across different geographic regions and clinical settings, thereby limiting their generalizability. Third, the model was evaluated using internal validation only; external validation in independent, multi-center cohorts is warranted to confirm its predictive robustness prior to any potential clinical application. Fourth, the primary outcome was restricted to binary seizure control, without incorporating longitudinal neurodevelopmental data—a critical dimension of IESS prognosis—owing to the absence of consistent psychomotor assessments in this retrospective cohort. Future research should prioritize multi-center, prospective designs to enhance the generalizability and translational relevance of the findings. Such studies should incorporate multidimensional long-term endpoints, including detailed seizure semiology, nuanced interictal EEG patterns, and standardized neurodevelopmental assessments. External validation across diverse populations remains essential to establish model robustness. A promising direction involves integrating the validated model into electronic health record (EHR) systems as a clinical decision support tool, enabling real-time, individualized prognostic assessment and therapeutic guidance for children with IESS at the point of care.

Furthermore, while several clinical factors closely associated with seizure outcomes have been identified, numerous other elements—such as detailed genetic background and molecular biomarkers—may significantly influence IESS prognosis but were not included in this analysis. Future investigations should seek to integrate more comprehensive genomic data and incorporate molecular biology profiling. The inclusion of multi-omics data, potentially covering genetics, transcriptomics, and proteomics, is expected to greatly enhance the accuracy, biological relevance, and comprehensiveness of predictive models for IESS. This integrated approach could ultimately lead to more precise prognostic stratification and the development of targeted therapeutic interventions.

Current research on IESS treatment, both domestically and internationally, primarily focuses on growth retardation and developmental delays in affected children (4). Thus, future therapeutic development should emphasize neuroprotective combination therapies aimed at improving neurodevelopmental outcomes, ultimately improving patients’ quality of life. Beyond pharmacological approaches like the KD and ACTH, early surgical evaluation should be considered for children with clear MRI abnormalities or poor response to ACTH therapy. Surgical interventions, such as corpus callosotomy and focal resection for IESS associated with unilateral or bilateral lesions, have become increasingly common in recent years (59).

## 5 Conclusion

In summary, this study successfully developed an efficient predictive model for seizure outcomes in Infantile Epileptic Spasms Syndrome (IESS) by integrating machine learning algorithms with comprehensive clinical data. The XGBoost-based model demonstrated superior performance in stratifying high-risk patients and shows significant potential for informing personalized treatment strategies. This capability is crucial for optimizing therapeutic effectiveness and improving long-term prognosis. While the model exhibited excellent performance in our cohort, future research should prioritize multi-center external validation to confirm its generalizability across diverse populations. Further optimization of its clinical implementation is necessary to enhance its practical utility. These steps are essential to advance the model into a reliable tool that can ultimately improve management and treatment outcomes for children with IESS.

## Data Availability

The original contributions presented in the study are included in the article/Supplementary Material, further inquiries can be directed to the corresponding author/s.
