# Supplementary material for: Development and validation of a machine learning model to predict prognostic outcomes in infantile epileptic spasms syndrome
Source: Front Pediatr. 2026 Apr 7;14:1777561. doi: 10.3389/fped.2026.1777561 (PMC13096072; doi:10.3389/fped.2026.1777561)
Supplement: Supplementary file 1 [file Table1.docx]

| **TABLE S1 Clinical characteristics among patients stratified by prognostic outcomes in the training set.** | | | | | |
| --- | --- | --- | --- | --- | --- |
| Characteristics | | Overall，N=261^1^ | Prognostic outcomes | | P |
|  |  |  | Good prognosis group(N=145) | Poor prognosis group(N=116) |  |
| **Gender（n , %）** | |  |  |  | 0.797 |
| Female | | 99（37.9%） | 54（20.7%） | 45（17.2%） |  |
| Male | | 162（62.1%） | 91（34.9%） | 71（27.2%） |  |
| **Duration of follow-up(day)** | |  |  |  | 0.42 |
| Median (IQR) | | 1145(679.0 , 1915.0) | 1220（702.0 , 1881.0） | 1065.5(618.75 , 2008.0) |  |
| **Age at onset of IESS (months)** | |  |  |  | 0.341 |
| Median (IQR) | | 5.0（3.0，7.0） | 5.0（4.0，7.0） | 5.0（3.0，7.0） |  |
| **Diagnosis lag (days)** | |  |  |  | 0.787 |
| Median (IQR) | | 31.0（13.0，71.5） | 32.0（15.0，65.0） | 29.5（12.25，93.25) |  |
| **Treatment lag (days)** | |  |  |  | 0.627 |
| Median (IQR) | | 33.0（15.0，86.0） | 34.0（15.5，71.5） | 32.0（14.0，99.0） |  |
| **Number of spasms at diagnosis** | |  |  |  | 0.025 |
| Median (IQR) | | 5.0（3.0-8.0） | 5.0（3.0，7.0） | 6.0（4.0，8.0） |  |
| **Etiology,n (%)** | |  |  |  | 0.045 |
| Unknown | | 155（59.4%） | 94（36.0%） | 61（23.4%） |  |
| Known | | 106（40.6%） | 51（19.5%） | 55（21.1%） |  |
| **Genetic** | | 59（22.6%） | 29（11.1%） | 30（11.5%） | 0.261 |
| **Structural** | | 65（24.9%） | 27（10.3%） | 38（14.6%） | 0.009 |
| **Metabolic** | | 5（1.9%） | 2（0.8%） | 3（1.1%） | 0.801 |
| **Perinatal abnormalities,** (n , %) | |  |  |  | 0.049 |
| Yes | | 74（28.4%） | 34（13.0%） | 40（15.3%） |  |
| No | | 187（71.6%） | 111（42.5%） | 76（29.1%） |  |
| **Family history, n (%)** | |  |  |  | 0.54 |
| Yes | | 10（3.8%） | 7（2.7%） | 3（1.1%） |  |
| No | | 251（96.2%） | 138（52.9%） | 113（43.3%） |  |
| **Developmental delay at onset of IESS, n (%)** |  | |  |  | 0.836 |
| Yes | | 176（67.4%） | 97（37.2%） | 79（30.3%） |  |
| No | | 85（32.6%） | 48（18.4%） | 37（14.2%） |  |
| **Developmental delay at the last follow-up, n (%)** |  | |  |  | 0.021 |
| Yes | | 235（90.0%） | 125（47.9%） | 110（42.1%） |  |
| No | | 26（10.0%） | 20（7.7%） | 6（2.3%） |  |
| **Neurologic examination at onset of IESS, (n , %)** |  | |  |  | 0.181 |
| Yes | | 66（25.3%） | 32（12.3%） | 34（13.0%） |  |
| No | | 195（74.7%） | 113（43.3%） | 82（31.4%） |  |
| **Seizure types（n , %）** | |  |  |  | ＜0.001 |
| Epileptic spasms only | | 188（72.0%） | 120（46.0%） | 68（26.1%） |  |
| Spasm with other seizure types | | 73（28.0%） | 25（9.6%） | 48（18.4%） |  |
| **Hypsarrhythmia（n , %）** | |  |  |  | 0.512 |
| Typical/Atypical Hypsarrhythmia | | 179（68.6%） | 97（37.2%） | 82（31.4%） |  |
| No Hypsarrhythmia | | 82（31.4%） | 48（18.4%） | 34（13.0%） |  |
| **MRI findings（n , %）** | |  |  |  | 0.006 |
| Normal | | 172（65.9%） | 106（40.6%） | 66（25.3%） |  |
| Abnormal | | 89（34.1%） | 39（14.9%） | 50（19.2%） |  |
| **MRI abnormalities-types** | |  |  |  |  |
| Encephalomalacia | | 47（18.0%） | 25（9.6%） | 22（8.4%） | 0.719 |
| Coexisting TSC | | 12（4.6%） | 5(1.9%) | 7(2.7%) | 0.322 |
| Neuronal Migration Disorder | | 13（5.0%） | 6(2.3%) | 7(2.7%) | 0.484 |
| Brain injury | | 6（2.3%） | 0 | 6(2.3%) | 0.019 |
| Brain Atrophy | | 7（2.7%） | 2(0.8%) | 5(1.9%) | 0.284 |
| Dysgenesis of corpus callosum | | 6（2.3%） | 2(0.8%) | 4(1.5%) | 0.489 |
| Tumor | | 1（0.4%） | 0 | 1(0.4%) | 0.911 |
| **Initiating treatment with ACTH（n , %）** | |  |  |  | 0.393 |
| Yes | | 175（67.0%） | 94(36.0%) | 81(31.0%) |  |
| No | | 86（33.0%） | 51(19.5%) | 35(13.4%) |  |
| **Response to ACTH therapy at day 14（n , %）** |  | |  |  | 0.005 |
| Effective | | 91（34.9%） | 60(23.0%) | 32(12.3%) |  |
| Ineffective | | 84（32.2%） | 35(13.4%) | 49(18.8%) |  |
| **Number of total ASMs, n (%)** | |  |  |  | ＜0.001 |
| Median (IQR) | | 4.0（2.0，5.0） | 2.0（2.0-3.0） | 5.0（4.0，7.0） |  |
| **Use of first-line ASMs（n , %）** | |  |  |  | 1 |
| Yes | | 153（58.6%） | 85(32.6%) | 68(26.1%) |  |
| No | | 108（41.4%） | 60(23.0%) | 48(18.4%) |  |
| **Initiating treatment with KD（n , %）** | |  |  |  | ＜0.001 |
| Yes | | 88（33.7%） | 27（10.3%） | 61（23.4%） |  |
| No | | 173（66.3%） | 118（45.2%） | 55（21.1%） |  |
| **Response to KD therapy（n , %）** | |  |  |  | ＜0.001 |
| Effective | | 28（10.7%） | 16（6.1%） | 12（4.6%） |  |
| Ineffective | | 60（23.0%） | 11（4.2%） | 49（18.8%） |  |
| **Surgery（n , %）** | |  |  |  | 0.323 |
| Yes | | 20（7.7%） | 9（3.4%） | 11（4.2%） |  |
| No | | 241（92.3%） | 136（52.1%） | 105（40.2%） |  |
| ^1^n (%); ^2^Wilcoxon rank sum test/Mann-Whitney U test; ^3^Pearson’s Chi-squared test; | | | | | |
| IESS: infantile epileptic spasm syndrome; TSC: Tuberous Sclerosis Complex;  ACTH: adrenocorticotropic hormone; KD: ketogenic diet; ASMs：Antiseizure medications; | | | | | |

| **Table S2 Univariable Cox regression analysis of children with IESS in the training set.** | | | |
| --- | --- | --- | --- |
| Characteristics | OR | 95% CI | P |
| Gender | 1.258 | 0.864-1.830 | 0.231 |
| Age at onset of IESS | 1.006 | 0.958-1.057 | 0.801 |
| Diagnosis lag | 1.000 | 0.998-1.001 | 0.787 |
| Treatment lag | 0.999 | 0.998-1.000 | 0.161^*^ |
| Number of spasms at diagnosis | 1.048 | 0.998-1.101 | 0.059^*^ |
| Etiology | 1.112 | 0.772-1.602 | 0.569 |
| Genetic | 1.340 | 0.897-2.002 | 0.153^*^ |
| Structural | 0.815 | 0.534-1.243 | 0.342 |
| Metabolic | 3.134 | 0.980-10.019 | 0.054^*^ |
| Perinatal abnormalities | 1.580 | 1.075-2.321 | 0.020^*^ |
| Family history | 0.348 | 0.110-1.101 | 0.072^*^ |
| Developmental delay at onset of IESS | 0.684 | 0.460-1.017 | 0.061^*^ |
| Developmental delay at the last follow-up | 0.568 | 0.250-1.294 | 0.178^*^ |
| Neurologic examination at onset of IESS | 1.284 | 0.860-1.917 | 0.221 |
| Seizure types | 1.485 | 1.023-2.155 | 0.038^*^ |
| Hypsarrhythmia | 1.095 | 0.733-1.634 | 0.658 |
| MRI findings | 0.951 | 0.640-1.413 | 0.804 |
| Encephalomalacia | 0.638 | 0.375-1.086 | 0.098^*^ |
| Coexisting TSC | 2.013 | 0.931-4.350 | 0.075^*^ |
| Neuronal Migration Disorder | 2.525 | 1.167-5.463 | 0.019^*^ |
| Brain injury | 1.430 | 0.452-4.530 | 0.543 |
| Brain Atrophy | 0.827 | 0.262-2.614 | 0.747 |
| Dysgenesis of corpus callosum | 0.565 | 0.139-2.291 | 0.424 |
| Tumor | 0.877 | 0.122-6.299. | 0.896 |
| Initiating treatment with ACTH | 1.990 | 1.320-2.999 | 0.001^*^ |
| Response to ACTH therapy at day 14 | 0.819 | 0.657-1.020 | 0.075^*^ |
| Number of total ASMs | 1.156 | 1.084-1.233 | ＜0.001^*^ |
| Use of first-line ASMs | 1.699 | 1.699-1.151 | 0.008^*^ |
| Initiating treatment with KD | 0.611 | 0.423-0.884 | 0.009^*^ |
| Response to KD therapy | 0.807 | 0.630-1.034 | 0.090^*^ |
| Surgery | 1.139 | 0.609-2.129 | 0.684 |
| ^*^p < 0.2; | | | |

| **Table S3 Performance metrics for six models in training dataset** | | | | | | |
| --- | --- | --- | --- | --- | --- | --- |
| Model | AUROC | accuracy | precision | recall | specificity | F1-score |
| LR | 0.815 [0.766-0.864] | 0.748 | 0.766 | 0.621 | 0.849 | 0.686 |
| RF | 0.808 [0.759-0.857] | 0.732 | 0.703 | 0.682 | 0.771 | 0.692 |
| SVM | 0.792 [0.740-0.843] | 0.711 | 0.717 | 0.576 | 0.819 | 0.639 |
| CART | 0.770 [0.715-0.824] | 0.765 | 0.709 | 0.795 | 0.741 | 0.75 |
| XGB | 0.827 [0.781-0.874] | 0.758 | 0.714 | 0.758 | 0.759 | 0.735 |
| LGB | 0.815 [0.767-0.863] | 0.732 | 0.678 | 0.75 | 0.717 | 0.712 |
| AUROC: Area under the receiver operating characteristic curve; LR: Logistic Regression; RF: Random Forest; SVM Support Vector Machine; CART Classification and Regression Tree; XGB: eXtreme Gradient Boosting; LGB: Light Gradient Boosting Machine; | | | | | | |
